# High Prevalence of *Pfhrp*2/3 Gene Deletions and Major Threat to Malaria Control Programs in Ethiopia

**DOI:** 10.1155/2024/8848997

**Published:** 2024-11-02

**Authors:** Sisay Getie, Gebeyaw Getnet Mekonnen, Aline Lamien Meda, Meseret Birhanie, Aberham Abere, Harald Noedl

**Affiliations:** ^1^Department of Medical Parasitology, School of Biomedical and Laboratory Science, College of Medicine and Health Science, University of Gondar, Gondar, Ethiopia; ^2^Institute for Specific Prophylaxis and Tropical Medicine, Medical University of Vienna, Vienna, Austria

**Keywords:** Aykel, Ethiopia, Negade Bahir, *Pfhrp*, *Pfhrp3*, *Plasmodium falciparum*, Sanja

## Abstract

**Background:** Rapid diagnostic tests (RDTs) targeting *pf*histidine-rich protein 2 (*Pfhrp*2) are widely used for diagnosis of *Plasmodium falciparum* infections in resource-limited malaria endemic countries. However, test results are affected by deletions of the *Pfhrp2*, *Pfhrp3*, and flanking genes and associated negative results from rapid diagnostic devices were previously reported. Therefore, the aim of this study was to reveal the existing genetic profile of *Pfhrp*2 and *Pfhrp*3 genes of *P. falciparum*-infected patients in northwestern Ethiopia.

**Methods:** A total number of 302 blood samples were collected from children at Chilga (Aykel, Negade Bahir), and Sanja health centers in northwestern Ethiopia. Thirty-three (10.9%) samples tested positive for *P. falciparum* malaria. The *Pfhrp*2, *Pfhrp*3, and flanking genes (MAL7P1_228 and MAL7P1_230 for *Pfhrp*2, and MAL13P1_475 and MAL13P1_485 for *Pfhrp*3) were amplified using standard nested-PCR.

**Results:** *Pfhrp*2 and both of its flanking genes were found to be present in 12 (36.4%) out of the 33 samples. Twenty-one (63.6%) samples tested negative for the *Pfhrp2* gene and 19 samples (57.6%) tested positive for at least one of the flanking genes. Five (15.2%) samples gave positive results for the *Pfhrp*3 gene and both of its flanking genes, whereas 16 (48.5%) tested negative for all three.

**Conclusions:** Our study provides widespread deletions in the *Pfhrp*2 and *Pfhrp*3 genes in Ethiopia, thereby confirming anecdotal reports of diagnostic failure with *Pfhrp*2-based RDTs in the region. The implications of our finding for the current diagnostic paradigm, which relies on the detection of *P. falciparum* by *Pfhrp*2-based RDTs in remote areas, may need rethinking.

## 1. Background

In the past decade, the world moved forward in the fight against malaria with the implementation of a combination of preventive and control measures including the use of long-lasting insecticide-treated nets (LLIN), community deployment of malaria rapid diagnostic tests (RDTs) and treatment using artemisinin combination-based therapies (ACT), and social behavioral change and communication. Despite global efforts, these numbers have only decreased modestly for the past 5 years [1]. Reasons underlying this include a higher number of *hrp2/3* deletions in *Plasmodium falciparum* that decreases the sensitivity in RDTs [2]. Despite this, according to the 2016 World Health Organization (WHO) report, there were 212 million cases and 429,000 deaths of malaria globally with about 90 percent of the cases and deaths occurring in sub-Saharan Africa [1].

Likewise, malaria is yet among the major public health problem in Ethiopia where *Plasmodium falciparum* and *Plasmodium vivax* are the main species accounting for roughly 63.6% and 36.4% of malaria cases. Over the last 10 years morbidity and mortality due to malaria decreases in Ethiopia with the implementation of scale-up universal intervention measures [3]. This effort enabled the country to set malaria elimination strategy with objectives to: reduce malaria case incidence to zero by 2030, reduce malaria mortality rate to zero by 2030 and prevent re-establishment of malaria in all historically malaria free districts [4].

However, one of the main challenges in combating malaria that need to be addressed is inadequate diagnostic capacity towards malaria. In Ethiopia, diagnosis of malaria is mainly relying on microscopic or RDTs with limitations of low sensitivity and problems of detection and identification of the *Plasmodium* species [5, 6].

In spite of this, RDTs is mainly used in the outskirt areas of the country where there is no access to microscopic diagnosis. Since 2011 the Federal Ministry of Health (FMOH) of Ethiopia has launched a health extension package accompanied with RDTs and a stock of anti-malaria treatments to be deployed in communities for early management and reduction of malaria [7]. In the growing effort of global malaria elimination strategies, WHO recommends countries to use RDTs to support mass screening and treatment of malaria depends on the effect that it brings. Currently available commercial malaria RDTs is mainly targeted to detect histidine-rich protein 2 (HRP2) in the case of *P. falciparum* and that of plasmodium lactate dehydrogenase (pLDH) for non-*falciparum* species [8, 9]. HRP2 is only expressed by *P. falciparum* encoded by *Pfhrp2* gene. The gene is located in chromosome 8 with flanking regions of MAL7P1_228 and MAL7P1_230 in the upstream and downstream of *Pfhrp2*, respectively. *P. falciparum* also expresses HRP3 protein, which is encoded by *Pfhrp3* gene in chromosome 13 [10, 11].

These antigens found in peripheral circulation of *P. falciparum* infected patients and detected by RDTs, which is tagged with nitrocellulose membrane where monoclonal anti-HPR2 antibody is coated [11]. Although this antibody is supposed to detect mainly HRP2 antigen, structural motif similarity between HRP2 and HRP3 contribute to cross reactivity of the antibody with HRP3 [12]. Recently, different studies across the world reported high prevalence of genetic deletion of *Pfhrp*2 and *Pfhrp*3 in *P. falciparum* species. A study from Colombia revealed that the proportion of *Pfhrp*2 gene deletion was 38.5% among *P. falciparum* tested positive samples while that of *Pfhrp*3 was 45.5%. In these samples, at least 90% of upstream and downstream flanking genes for both *Pfhrp*2 and *Pfhrp*3 were also deleted. All these samples were tested negative by malaria RDTs although parasitaemia density was equal or above 500 parasites/*μ*L [13]. A study from Peru had also reported that eight among nine *P. falciparum* tested positive samples had *Pfhrp2* and *Pfhrp*3 genes deletion and ELISA negative result for HRP2 and HRP3 proteins [14]. Whereas a study from Ghana presented that about 36.2% of confirmed *P. falciparum* positive samples had *Pfhrp*2 gene deletion [15]. Polymorphism of HRP2 antigens and alteration of antibody recognition sites is also another challenge in the use of RDTs for the diagnosis of malaria [16–18].

Field performance of malaria RDTs, therefore, is not only about the quality of the kits but also the genotypes of the parasite. WHO puts endeavors to maintain the quality of RDTs and suggests countries to procure only WHO prequalified RDTs, to create a national legal framework for batch testing and quality control activities of RDTs and to investigate suspected false negative RDTs results [2, 9].

Although contradicting results of RDTs as compared to microscopy and molecular techniques have been reported in Ethiopia, information on the current genotype status of *Pf*hrp*2* and *Pf*hrp*3* in *P. falciparum* is not known in the country. Therefore, it is much worth important to reveal the existing genetic profile of *Pfhrp*2 and *Pfhrp3* genes of *P. falciparum* from infected patients to provide baseline data about the use of RDTs in Ethiopia to support the country's malaria elimination strategic plan.

## 2. Methods

### 2.1. Study Design, Period, and Area

**Figure 1:**
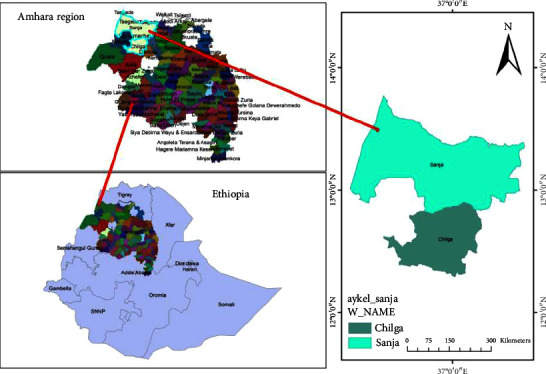
Map of the study area in North Gondar, Amhara Region, Ethiopia. Chilga (Negade Bahir and Aykel), and Sanja Health Center represents the geographical area were the study was conducted.

This study is an extension of a cross-sectional study conducted from March to November 2015 at Chilga (Aykel, Negade Bahir) and Sanja Health Centers in northwest Ethiopia where approximately over 100 thousand people get health service at these health facilities. Malaria is believed to be endemic in these areas of the country (Figure 1) [19].

### 2.2. Study Subjects

Children attended to the pediatrics out patient's department (OPD) at the Health Centers with clinical signs and symptoms consistent with malaria were included. The study participants were recruited continuously using convenient sampling method.

#### 2.2.1. Dry Blood Spot Sample Collection and Molecular Detection *P. falciparum*

Two drops of finger prick blood spots were collected aseptically from each participant and transferred onto filter paper (Whatman #903, GE Healthcare) labeled with the participant's study code and date. After each filter air-dried individually, they stored in small sealable plastic bags and transported to the Institute of Specific Prophylaxis and Tropical Medicine, Medical University of Vienna (MUV), Vienna, Austria, for molecular analysis. Genomic DNA was extracted from all dry blood spots collected using available genomic extraction kit. Malaria parasitaemia and *P. falciparum* species were confirmed by detecting gene of 18S ribosomal RNA using nested polymerase chain reaction (PCR) [20].

### 2.3. Genetic Detection of *Pfhrp*2, *Pfhrp*3, and Their Flanking Genes

Once genomic DNA is extracted from Dry blood spot sample using QIAamp mini-DNA kits (QIAGEN, https://www.qiagen.comExternal Link) according to the manufacturer's directions. Presence of *Pf*hrp2 and *Pf*hrp3 (exon 1, the intron, and exon 2) and flanking genes (MAL7P1_228 and MAL7P1_230 for *Pfhrp2*, and MAL13P1_475 and MAL13P1_485 for *Pfhrp*3) was confirmed by standard nested-PCR amplification as described previously [21].

## 3. Results

### 3.1. Study Identification and Selection

**Figure 2:**
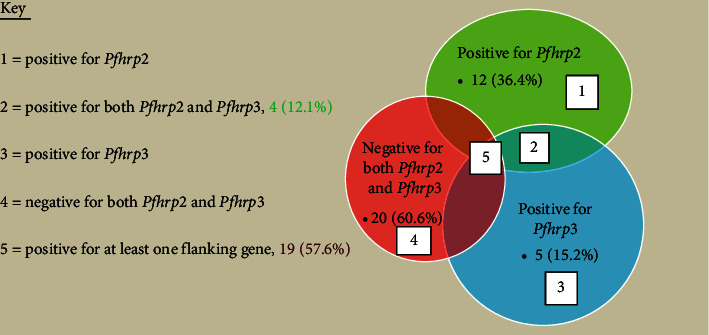
Venn's diagram *hrp*2/3 and franking genes both or either one deletions.

Out of the total 302 samples analyzed 33 (10.9%) were tested *P. falciparum* positive by nested-PCR. By the virtue its biology, *P. falciparum* is the only *Plasmodium* species that expresses *Pfhrp*2 and *Pfhrp*3 proteins, which are found in the blood circulation of *P. falciparum*-infected patients and are used to diagnosis falciparum malaria using rapid diagnostic devices. The normal expression of *Pfhrp*2 and *Pfhrp*3 proteins requires the presence of *Pfhrp*2 and *Pfhrp*3 genes and the respective flanking regions, respectively, in inframe position. Our study sought to provide baseline data on the genetic profile of *Pfhrp*2*, Pfhrp*3, and their flanking genes in *P*. *falciparum* positive samples. Among 33 *P. falciparum* positive samples, *Pfhrp*2 and both of its flanking genes, MAL7P1.230 and MAL7P1.228, were found to be present in only 12 (36.4%). Twenty-one (63.6%) samples tested negative for the *Pfhrp*2 gene and 19 samples (57.6%) tested positive for at least one of the flanking genes. Two samples (6.1%) tested negative for both *Pfhrp*2 and both of its flanking genes. Only 4 (12.1%) out of the 33 samples identified positive for both the *Pfhrp*2 and *Pfhrp*3 genes whereas 20 (60.6%) samples tested negative for both the *hrp*2/3 genes (Figure 2).

Only 5 (15.2%) samples gave positive results for the *Pfhrp*3 gene and both of its flanking genes, whereas 16 (48.5%) tested negative for all three and 12 (36.4%) for *Pfhrp*3 and at least one of the flanking genes (Table 1).

Among the three health centers in the study area, two *P. falciparum* positive samples (6.1%) from Aykel found to contain both the *Pfhrp*2 and *Pfhrp*3 and the respective flanking genes. *Pfhrp*2 and its flanking genes were found to present in 7 (21.2%) falciparum positive samples whereas that of *Pfhrp*3 and its flanking genes found in 2 (6.1%) samples from Negade Bahir. Lastly, the *Pfhrp*2 and its flanking genes present in 3 (9.1%) samples and that of *Pfhrp*3 and its flanking genes present in 1 (3.0%) sample from Sanja health center (Table 2).

## 4. Discussion

Malaria RDTs device is the mainstay of malaria diagnosis, and are sought to support malaria elimination strategies through community mass-screening [9, 22], in resource limited malaria-endemic world such as sub-Saharan African regions, where *P. falciparum* is the most common cause of illness and death [1]. Malaria HRP2-based RDTs target to detect HRP2 antigen, which is encoded by *Pfhrp2* gene of all *P. falciparum* species and is secreted into peripheral circulation of falciparum-infected patients. Therefore, a defect happen in the *Pfhrp2* gene affects the production of normal HRP2 antigen. Recent data from Latin America suggest deletions of *Pfhrp2* and both of its flanking genes and associated negative results from RDTs device in the diagnosis of falciparum malaria [13, 14].

Our study revealed deletion of *Pfhrp2* gene in 63.6% (21/33) samples and deletion of *Pfhrp3* gene in 84.9% (28/33) samples. This supports an increasing widespread of deletion of *Pfhrp2* and *Pfhrp3* genes of *P. falciparum* in Africa, where it is being reported most recently. Similar studies in the East African countries such as Eritrea, and Ethiopia revealed a 9.7% and 43%, and 27.3% and 30.5% deletion of *Pfhrp2* and *Pfhrp3* genes, respectively, in falciparum-positive patient samples [23, 24]. Another study in Djibouti 68.6% [25] 86.5% [26] *Pfhrp2*/*Pfhrp3* gene deletions report.

Among a limited number of samples in our study, we reported a higher proportion of deletion of both *Pfhrp2* and *Pfhrp3* genes than any other similar studies in East African countries, supporting a previous study in the same region of the country presenting a low sensitivity of RDTs to detect *P. falciparum* malaria as compared to molecular techniques [5]. Although our study did not provide RDTs results for the entire samples, low sensitivity of RDTs could be contributed from false negative results. Given that false-negative results of RDTs could be due to several factors such as storage condition of the device, RDTs lot type, wrong interpretation of the results, parasite density, and genotype of the malaria parasite and polymorphism of the antigens to be detected [12, 15, 27, 28].

Although RDTs handling and technical errors occur while working with RDTs would be improved through retraining, false-negative results due to alteration of genotype of the malaria parasite may be challenging. The implication is therefore all false–negative results from RDTs device need to be investigated in-depth for the essence of malaria elimination and monitoring programs.

Since the detection of HRP2 protein in the plasma of patients infected with falciparum malaria is confirmed [29, 30], HRP2-directed diagnosis and treatment of falciparum malaria has become common. The fact that HRP2 protein is important in the pathogenesis of *P. falciparum* [31], and has been suggested to prevent parasite intoxication from hemozoin [32], deletion of *Pfhrp2* gene has been reported to occur independently among all *P. falciparum* species.

Although there is no clear evidence demonstrating the root-cause of deletion of this gene, frequent use of HRP2-based RDTs for the diagnosis and treatment of malaria has been suggested to create selective pressure to the *Pfhrp2* gene [11, 33]. Taking the role of HRP2 in the detoxification of heme for the survival of the parasite and the mode of action of coartem on heme during the treatment of falciparum malaria in to consideration [34], presumably continuous use of coartem may induce pressure on *Pfhrp2* gene. RDTs device and coartem are introduced in 2004 into Ethiopia and are widely used in the diagnosis and treatment of falciparum malaria, respectively [35].

This may comply with a high prevalence of *Pfhrp2* gene deletion in Ethiopia as in our study. Although the first report of deletion of *Pfhrp2* gene has come out from Latin America in 2010 [14], the origin of deletion of *Pfhrp2* gene has been suggested to be from multiple clonal lineages and supposed to be dated as back as 1998 [11]. In this regard, we are not sure of the origin of deletion of *Pfhrp2* gene in Ethiopia in our study. Given that both HRP2 and HRP3 antigens are detected in peripheral circulation of falciparum infected patients, and have structural motif similarity [12], RDTs positivity was reported in the presence of only *Pfhrp3* regardless of *Pfhrp2* gene [36, 37].

However, it has been suggested that the presence of high parasitaemia level is detrimental for RDTs positivity. Our study revealed only 9 (27.3%) samples which contain at least either *Pfhrp2* or *Pfhrp3,* with high proportion of deletion of *Pfhrp3* gene. The fact that the locus of *Pfhrp2* and *Pfhrp3* genes is in different chromosomes of *P. falciparum*, their expression seems to be independent to each other. From this point, these genes seem to be evolved independently to attribute both genetic polymorphism and gene deletion.

However, according to recent genetic studies of *P. falciparum* across the world, deletion of both genes simultaneously at different rate seems to be unclear. A significant number of P. falciparum parasites in Ethiopia, have been found to lack the HRP2 gene [38]. This mutation leads to the absence of HRP2 antigen production, preventing the detection of these parasites in patients' blood using RDTs that are designed specifically for HRP2. HRP2-based RDTs are widely recognized as the most sensitive, stable, and commonly utilized tests for diagnosing *P. falciparum* malaria.

Our study provides clear evidence of widespread deletions in the *Pfhrp2* and *Pfhrp3* genes in Ethiopia, thereby confirming anecdotal reports of diagnostic failure with HRP2-based RDTs in the region. The implications of our findings for the existing diagnostic framework, which depends on HRP2-based RDTs to detect *P. falciparum* in remote regions, may require reevaluation. Initial prospective surveys investigating the deletion of the HRP2 and HRP3 genes in *P. falciparum* populations have been completed or are nearing completion in Ethiopia.

One of the limitations of our study is that RDTs test was not performed for all the samples analyzed for molecular genotyping of the *Pfhrp2* and *Pfhrp3* and the respective flanking genes and, therefore, performance of locally available RDTs to these samples is not obtained.

## Figures and Tables

**Table 1:** Frequency of *Pfhrp*2 and *Pfhrp*3 and their flanking genes by PCR amplification among falciparum positive samples in northwest Ethiopia.

MAL7P1_228	MAL7P1_230	*Pfhrp*2 exon 1–2	All Pf. samples	MAL13P1_475	MAL13P1_485	*Pfhrp*3 exon 1–2	All Pf. samples	*Pfhrp*2 exon 1–2	*Pfhrp*3 exon 1–2	All Pf. samples
1	1	1	12	1	1	1	5	1	1	4
1	1	0	5	1	1	0	0	1	0	8
1	0	0	14	1	0	0	2	0	1	1
1	0	1	0	1	0	1	0	0	0	20
0	0	1	0	0	0	1	0			
0	1	0	0	0	1	0	10			
0	1	1	0	0	1	1	0			
0	0	0	2	0	0	0	16			
Total			33				33			33

*Note:* Where 1 = presence; and 0 = absence of a gene in the parasite genome.

**Table 2:** Frequency of PCR amplification of *Pfhrp*2 and *Pfhrp*3 and their respective flanking genes among *falciparum* positive samples, by health center site, in northwest Ethiopia.

Sample id	Nested PCR/Pf	MAL7P1_228	MAL7P1_230	*Pfhrp*2 exon 1–2	MAL13P1_475	MAL13P1_485	*Pfhrp*3 exon 1–2
AK12	1	1	1	1	1	1	1
AK23	1	1	1	0	0	1	0
AK26	1	1	0	0	0	0	0
AK60	1	1	1	1	1	1	1
NB06	1	1	1	1	0	1	0
NB26	1	1	1	1	1	1	1
NB43	1	1	0	0	0	0	0
NB60	1	1	0	0	0	1	0
NB62	1	1	0	0	0	0	0
NB69	1	1	0	0	0	0	0
NB71	1	1	1	1	0	1	0
NB73	1	1	1	1	0	1	0
NB74	1	1	0	0	0	1	0
NB76	1	1	1	1	1	1	1
NBL1	1	1	0	0	1	0	0
NBL2	1	1	1	1	0	1	0
NBL10	1	1	1	0	1	0	0
NBL23	1	0	0	0	0	0	0
NBL46	1	1	1	1	0	1	0
SG1821	1	1	1	1	0	0	0
SG1825	1	1	1	1	0	1	0
SG1829	1	1	0	0	0	0	0
SG1868	1	1	1	0	0	0	0
SG1873	1	1	0	0	0	0	0
SG1883	1	1	0	0	0	0	0
SG1887	1	0	0	0	0	0	0
SG2504	1	1	0	0	0	0	0
SG9215	1	1	1	0	0	0	0
SG9263	1	1	1	1	0	1	0
SG9273	1	1	0	0	0	0	0
SG9460	1	1	0	0	0	0	0
SG9465	1	1	0	0	0	0	0
SG9467	1	1	1	0	1	1	1
Total	33	31	17	12	7	15	5

*Note:* Where 1 = presence; and 0 = absence of a gene in the parasite genome.

## Data Availability

Data are available upon request.

## Nomenclature

ACT: Artemisinin combination-based therapies
FMOH: Federal Ministry of Health
HRP: Histidine rich protein
LLIN: Long-lasting insecticide-treated nets
MUV: Medical University of Vienna
nested PCR: nested polymerase chain reaction
OPD: Out patient's department
pLDH: Plasmodium lactate dehydrogenase
RDTs: Rapid diagnostic tests
WHO: World Health Organization
